# Preventing Postpericardiotomy Syndrome: Current Evidence and Future Directions

**DOI:** 10.3390/jcdd13020063

**Published:** 2026-01-24

**Authors:** Christos E. Ballas, Thomas Theologou, Evangelia Samara, Fotios Barkas, Theodora Bampali, Kyriakos Kintzoglanakis, Christos Diamantis, Petros Tzimas, Christos S. Katsouras, Christos Alexiou

**Affiliations:** 1 Department of Cardiac Surgery, University Hospital of Ioannina, 45500 Ioannina, Greece; 2 Department of Anesthesiology and Postoperative Intensive Care, University Hospital of Ioannina, 45500 Ioannina, Greece; 3 Faculty of Medicine, School of Health Sciences, University of Ioannina, 45500 Ioannina, Greece; 4 Cardiology Department, Hatzikosta General Hospital, 45445 Ioannina, Greece; 5 Local Health-Team Unit of Thebes, 32200 Boeotia, Greece; 6 Department of Primary Care, General Hospital of Livadeia, 32100 Livadeia, Greece; 7 First Department of Cardiology, University Hospital of Ioannina, 45500 Ioannina, Greece

**Keywords:** postpericardiotomy syndrome, prevention, colchicine, pericardial closure, biomarkers, cardiac surgery

## Abstract

Postpericardiotomy syndrome (PPS) is the most frequent inflammatory after-effect of cardiac surgery and is characterized by high morbidity, delayed hospitalization, and increased long-term mortality rates. Although PPS is common, empirical anti-inflammatory therapy has historically been employed for its prevention, and mechanism-based approaches have not yet been standardized. In this literature review, which was conducted on the basis of randomized controlled trials, meta-analyses, cohort studies, and mechanistic research regarding pharmacologic interventions, surgical modalities, and biomarker-based preventive strategies, the deficiencies of a critical synthesis of existing preventive strategies and emerging risk stratification instruments for PPS are addressed. The review affirms that the most evidence-based pharmacologic intervention is colchicine, which demonstrates a consistent reduction in PPS incidence across a range of randomized trials. Nonsteroidal anti-inflammatory drugs show variable responses, whereas corticosteroids are no longer recommended for routine prophylaxis due to relapse. Specific anti–interleukin-1 therapies represent a promising novel approach for high-risk patients. Surgical interventions, such as pericardial closure using biomaterials and posterior pericardiotomy, are important and do not lead to increased hemodynamic complications, while postoperative effusions, atrial fibrillation, and tamponade are reduced. Less invasive methods may also be employed to mitigate inflammatory causes, particularly in valve-sparing procedures and congenital operations. Emerging biomarker data, including postoperative neutrophil-to-lymphocyte ratios, C-reactive protein levels, and pericardial fluid cytokines, enable the identification of high-risk patients and form the basis for a personalized prevention approach. In summary, pharmacologic prophylaxis, innovative surgical techniques, and biomarker-based risk stratification represent a pathway toward reducing the incidence and burden of PPS in modern cardiac surgery.

## 1. Introduction

Postpericardiotomy syndrome (PPS) is one of the most common inflammatory complications of cardiac surgery, with an incidence reported to range from 9% to over 21%, depending on the patient population, type of surgery, and diagnostic criteria [1,2]. The syndrome is defined by the occurrence of fever, pleuropericardial effusions, chest pain, and elevated inflammatory markers, which usually appear days to weeks after surgery [3].

Despite the fact that the pathogenesis of PPS has not been fully elucidated, it is currently considered an autoimmune process that is initiated by surgical trauma, the exposure of pericardial and pleural antigens, and the subsequent activation of both innate and adaptive immunity [4,5]. Cardiac surgery, in addition to the systemic inflammatory reaction, also causes a local inflammatory response, according to studies showing increased accumulation of inflammatory cytokines in the pericardial fluid of postoperative patients (compared to peripheral blood). This means that the pericardial space functions as an autonomous inflammatory compartment after pericardiotomy. This combined local and systematic inflammatory response may lead to clinically significant effusions, cardiac tamponade, atrial arrhythmias, and prolonged recovery [6,7].

Recent studies have provided important data about the local inflammatory state of the pericardial space after cardiac surgery. Proteomic and cytokine profiling of pericardial fluid (postoperatively) demonstrates marked increases in multiple pro-inflammatory mediators. Those mediators include interleukins, interferon-γ, adhesion molecules, chemokines such as monocyte chemoattractant protein-1, and matrix metalloproteinases. The levels of those increases exceed those seen systemically, reflecting a compartmentalized inflammatory response. This is distinct from the peripheral circulation. This intense local inflammatory response is increasingly recognized as the main contributor to pleuropericardial effusions, postoperative arrhythmias, and the pathophysiology of PPS, and it underpins the growing interest in pericardial biomarkers for risk stratification and targeted therapy [7,8].

The clinical significance of PPS cannot be overstated, as it affects postoperative morbidity and healthcare resource utilization. Lehto et al. demonstrated that PPS is associated with a 1.78-fold increase in mortality, while severe cases requiring invasive intervention after aortic valve replacement were associated with a doubled risk of death (HR 2.01) [9,10]. Recurrence of PPS is also common, occurring in up to 38% of patients [11]. Despite its frequency and clinical impact, PPS has long been an underrecognized aspect of clinical practice [1].

Preventive measures have changed over the last 20 years from empiric anti-inflammatory therapy to more risk-adapted and mechanism-based methods. Colchicine represents the most evidence-based pharmacological prophylactic strategy, having been shown to reduce PPS incidence by 40–60% in multiple randomized trials and meta-analyses [12,13,14]. Conversely, corticosteroids are no longer regarded as a first-line prophylactic option because of their higher recurrence rates, and nonsteroidal anti-inflammatory drugs (NSAIDs) show inconsistent preventive efficacy [15,16,17]. Additionally, postoperative effusions and atrial fibrillation have been significantly diminished as a result of surgical innovations, including posterior pericardiotomy and pericardial closure with biomaterials [18,19,20].

Finally, there is growing interest in biomarker-based risk stratification, whereby systemic inflammatory indices [(e.g., Neutrophil-to-Lymphocyte Ratio (NLR), C-Reactive Protein (CRP)] and pericardial fluid biomarkers [(e.g., Interleukin (IL)-6, natriuretic peptides)] may be used to identify patients at high risk for PPS [21,22]. These developments provide the foundation for individualized preventive strategies that integrate pharmacological, surgical, and biomarker-guided approaches.

This review aims to summarize the existing evidence on pharmacological and surgical prevention of PPS and to highlight the emerging role of biomarkers in personalized preventive strategies. By doing so, it seeks to bridge the gap between traditional practices and modern, risk-adapted preventive approaches in contemporary cardiac surgery.

## 2. Pharmacological Interventions

### 2.1. Colchicine

Colchicine was traditionally regarded as the main pharmacological agent of preventing PPS. Its action is based on inhibiting the polymerization of microtubules and the migration of neutrophils as well as more recent data indicate that it also disrupts the activation of the NLR family pyrin domain containing 3 (NLRP3) inflammasome, thereby limiting the production and maintenance of interleukin-1b production and propagation of the inflammatory cascade [4]. Initial interest in its use was driven by observational data and small randomized trials, with the pivotal breakthrough provided by the Colchicine for the Prevention of the Postpericardiotomy Syndrome (COPPS) trial. This multicenter randomized controlled study demonstrated that postoperative colchicine reduced the incidence of PPS from 21.1% in the placebo group to 8.9% in the colchicine group, corresponding to a relative risk reduction of 57% [12].

The next COPPS-2 trial refined this approach by initiating colchicine preoperatively to enhance its prophylactic effect. Although the trial demonstrated a reduced incidence of PPS, it also reported a higher rate of gastrointestinal intolerance, leading to treatment discontinuation in 21% of patients receiving colchicine compared with 5% in the placebo group [17]. These results are supported by meta-analytical data. Agarwal et al., pooled data from randomized clinical trials and showed that colchicine reduced the PPS incidence at one year (13.2% vs. 25.8% with placebo; risk ratio 0.56, 95% CI 0.42–0.76), but with a more significant increase in adverse events (diarrhea, 12.5% vs. 8.5%, respectively) [13]. These quantitative data underscore the role of colchicine as the only pharmacological agent supported by robust randomized evidence for the prevention of PPS.

The beneficial effect of colchicine in preventing recurrent pericarditis has also been documented in clinical reports, supporting its broader use across the spectrum of post–cardiac injury syndromes [23]. However, tolerability issues—particularly gastrointestinal adverse effects—remain a significant limitation in clinical practice. Nevertheless, colchicine remains the only pharmacological agent officially recommended for PPS prophylaxis in international guidelines and represents a well-established and highly relevant preventive intervention.

Recent research has increasingly converged on colchicine as the most appropriate pharmacological intervention for the prevention of PPS. The extensive meta-analysis by Verma et al. with 15 randomized controlled trials and more than 3400 patients showed a relative decrease in PPS incidence by 50% and a considerable reduction in peri-procedural atrial fibrillation rates, but at the cost of gastrointestinal intolerability, necessitating drug discontinuation in approximately 10% of patients [14]. Similarly, the randomized trial by Mashayekhi et al., which included 240 participants, compared colchicine with placebo and demonstrated that colchicine reduced PPS incidence by nearly half (12.1% vs. 21.6%), indicating a preventive effect without serious complications [24]. Conversely, findings of efficacy in all studies have not been consistent. Both Amoli et al. and Meurin et al., showed that colchicine does not substantially decrease volumes of postoperative pericardial effusions, suggesting that its preventive effect is likely limited to inflammation-induced PPS rather than non-inflammatory effusions [25,26]. A recent meta-analysis by Lutschinger et al., supported these findings, showing that PPS incidence was reduced by 43%, with a number needed to treat of 10, while no benefit was observed with late administration after the first postoperative week [27]. Lastly, the recent study by Pan et al., demonstrated the benefit of low-dose regimens and revealed that colchicine did not only reduce the incidence of PPS (3.1% vs. 17.7%, among patients receiving colchicine therapy compared with those who did not, respectively) but also reduced markers of myocardial injury and systemic inflammation, providing further evidence of its pleiotropic cardioprotective effects [28].

Table 1 summarizes the key randomized controlled trials and meta-analyses evaluating the efficacy and effect estimates of colchicine for the prevention of PPS.

### 2.2. Nonsteroidal Anti-Inflammatory Drugs

NSAIDs that include ibuprofen, indomethacin, and diclofenac have historically been the mainstay of symptomatic treatment for PPS. Their prophylactic use, however, has remained controversial.

Rabinowitz et al. conducted a large retrospective study in a pediatric population to evaluate the effect of prophylactic ibuprofen on the prevention of PPS following surgical atrial septal defect repair. Among the 207 patients included, the overall incidence of PPS was 10%, with no significant difference between those who received ibuprofen and those who did not (*p* = 1.0). Notably, none of the patients who required pericardiocentesis had received ibuprofen prophylaxis, further suggesting that prophylactic ibuprofen had no impact on the incidence of severe PPS [16]. Overall, this study demonstrated the lack of efficacy of ibuprofen as a prophylactic measure in pediatric congenital heart surgery.

Conversely, evidence supporting a potential benefit of diclofenac has been reported. Sevuk et al. conducted a retrospective study comparing 100 patients who received diclofenac after cardiac surgery with 100 control patients. Diclofenac was associated with a marked reduction in PPS incidence (20% vs. 43%, *p* < 0.001) as well as a lower risk of pericardial effusion (15% vs. 30%, *p* = 0.01). Logistic regression analysis identified diclofenac administration as a protective factor against PPS [odds ratio (OR) 0.34, 95% confidence interval (CI) 0.18–0.65] [15]. These findings suggest heterogeneity in NSAID efficacy, with diclofenac potentially being more effective than ibuprofen for PPS prevention.

Overall, NSAIDs remain widely used for the treatment of PPS, while their preventive action remains a controversial issue. Other reviews, including that by Malik et al., have concluded that NSAIDs are effective for symptom control and the management of PPS recurrences, but that their prophylactic use is not supported by strong evidence [29]. In this context, colchicine appears to be the more effective preventive option, whereas NSAIDs may still have a role within combination regimens, particularly in patients in whom colchicine is contraindicated or poorly tolerated.

### 2.3. Corticosteroids

The history of corticosteroid use in the prevention of PPS is long and elaborate. Initial investigations indicated some benefit in reducing the prevalence of PPS and shortening the duration of symptoms. However, subsequent evidence has demonstrated significant limitations, particularly regarding an increased risk of recurrence and other adverse consequences. In reviews of prospective cohorts, Lehto and Kiviniemi pointed out that corticosteroid therapy has consistently been associated with increased recurrent pericarditis [17]. Malik et al. also highlighted that corticosteroids, although effective in symptom control, offer no long-term prognostic benefit and may even exacerbate recurrence [29].

This counterintuitive response can be attributed to the immunosuppressive effect of corticosteroids, which, in suppressing acute inflammation, can undermine the normal resolution step of the autoimmune process that causes PPS. Abadie and Cremer also highlighted that corticosteroid therapy is related to addiction and higher risk of recurrence, particularly when used in high doses or tapered at a rapid rate [18]. The totality of the evidence thus discourages their use as a prophylactic measure. The use of corticosteroids is now widely suggested as an alternative to NSAIDs and colchicine use when patients have contraindications; these include patients with renal dysfunction, pregnancy, or intolerance.

Quantitative benefit is lacking, and there is a risk of continuing disease activity, which restricts the usefulness of corticosteroids to a second-line option in PPS prophylaxis. This agreement represents a significant change compared to previous decades, which indicates a change in the knowledge of the autoimmune pathogenesis of PPS.

### 2.4. Novel and Targeted Therapies

The past few years have seen the development of biologic agents that block certain inflammatory mechanisms, especially interleukin (IL)-1 blockade. The clinical trials of IL-1 antagonists are a result of progress in the field of recognition of the central role of the NLRP3 inflammasome and IL-1b in pericardial inflammation. Vecchio et al. revealed that IL-1 is a central mediator of the autoimmune response in pericarditis, which offers a mechanistic explanation of the effect of anakinra, canakinumab, and rilonacept on the treatment [4].

Anakinra, an IL-1 receptor antagonist recombinant, has demonstrated a stable efficacy in pericarditis with corticosteroid dependence and colchicine resistance. Imazio et al. conducted a review of randomized trials and observational studies, indicating that the daily injection of anakinra as subcutaneously as possible resulted in the quick control of symptoms and the decreased frequency of recurrences, but the non-adherence rates were not more than 4%. Rilonacept, a soluble decoy receptor to IL-1a and IL-1b, has also shown superiority to placebo in randomized controlled trials, where, based on dosing schedule, weekly dosing has an advantageous advantage [30].

Monoclonal antibody against IL-1b (canakinumab) is less studied, and the effects of this agent have been contradictory in case reports. Interestingly enough, Signa et al. have mentioned two cases in children in which canakinumab was not effective in preventing recurrences, but both children reacted positively to anakinra. This implies that IL-1a could also be relevant in pathogenesis, which might be the reason why IL-1b selective blockade has limited effects [31].

**Figure 1:**
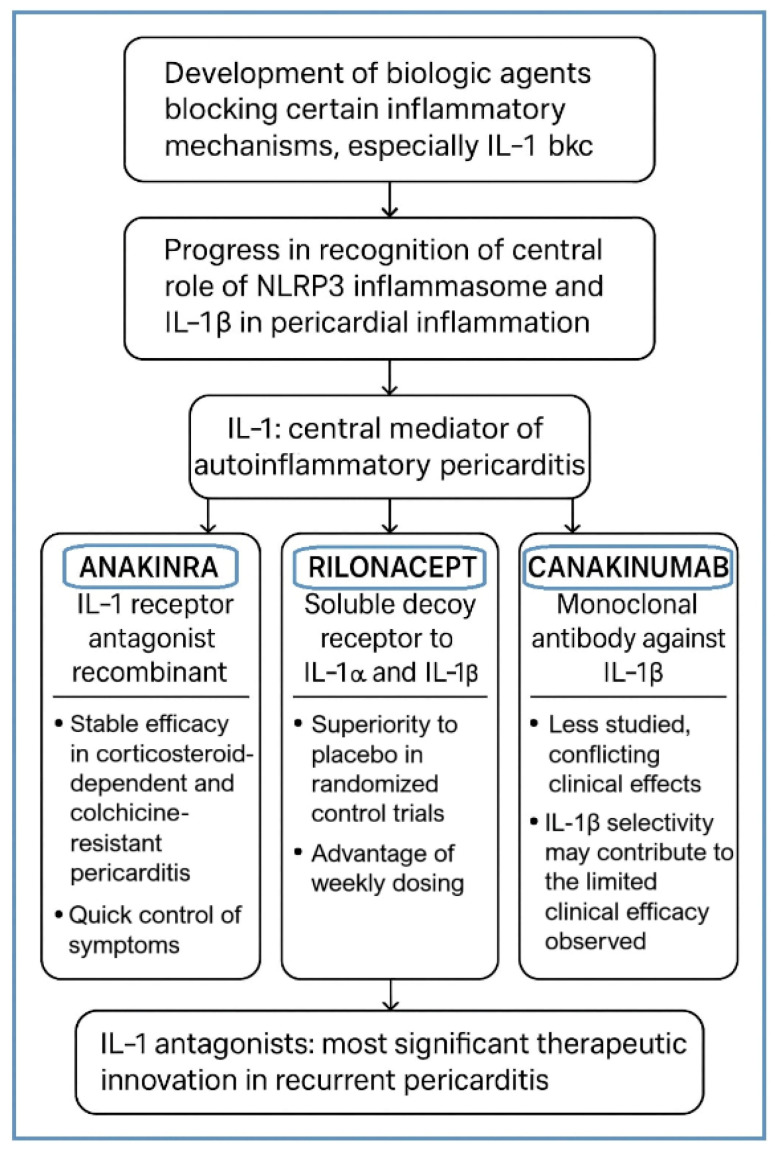
Pharmacologic agents targeting specific inflammatory pathways in pericarditis which are currently indicated for the treatment of recurrent disease rather than for preventive use. bkc: Blockade, IL: Interleukin, NLRP3: NLR family pyrin domain containing 3.

According to Abadie and Cremer, IL-1 antagonists are the most significant therapeutic innovation in recurrent pericarditis in recent years. They provide a solution to acute episodes in a short period of time and effective long-term control, particularly those that are not responsive to standard treatment [18]. Their use is not yet accepted as a primary mode of PPS prevention following surgery, but its application is growing, and additional trials could make them a prophylaxis in high-risk patients (Figure 1).

To date, these agents have been evaluated primarily in patients with established or refractory disease, often after failure or intolerance of conventional anti-inflammatory therapies. There is currently insufficient evidence to support their routine use in the prophylactic setting, and no large randomized trials have assessed their efficacy or safety for primary prevention of PPS.

## 3. Surgical and Procedural Strategies

### 3.1. Pericardial Closure Versus Non-Closure Techniques (Figure 2)

Among the most discussed surgical questions that arise in the prevention of PPS is whether the pericardium should be reconstructed or if it is better to leave it open following cardiac surgery. In the past, most surgeons preferred non-closure to prevent the hemodynamic impairment of postoperative myocardial swelling. But there is increasing evidence that pericardial reconstruction decreases adverse outcomes (Table 2). As found by Rego et al., pericardial closure is a procedure that has not been employed in the majority of cardiac operations in the United States (less than 20 percent) but that is now recommended by consensus experts as a standard intervention in the appropriate patient. The benefits reported are a reduction in pericardial adhesions, postoperative effusions, atrial fibrillation and bleeding complications, shorter hospital stays, and reduced readmissions [19].

**Figure 2:**
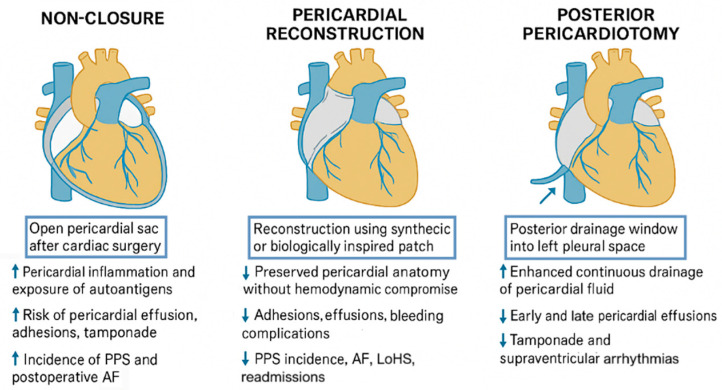
The benefits of pericardial reconstruction and posterior pericardiotomy compared with non-closure of the pericardium. PPS: Postpericardiotomy Syndrome, AF: Atrial Fibrillation, LoHS: Length of Hospital Stay.

Randomized trials in the same survey showed no result of increased adverse hemodynamics when synthetic substitutes or biologically inspired ones were employed in reconstructing the pericardium, as compared to using tight close-ups. Previous trials had demonstrated higher filling pressures when using native tissue closures, whereas current patch-based methods do not suffer this issue [19]. Husain et al. suggested a simple and repeatable technique of pericardial approximation by the application of hemostatic clips to the thymic fascia. This method took just a minute of extra operative time and gave safe securities to bypass grafts and great vessels but allowed safe re-entry in the case of reoperation. This method showed no early postoperative complications like tamponade, and it was found to have a lower occurrence of pericardial adhesions and possibly PPS [32].

Pericardial closure has demonstrated clinical benefit in observational cohort studies. Lehto et al. evaluated a cohort of 688 patients undergoing coronary artery bypass grafting (CABG) and reported a PPS prevalence of 8.9% requiring medical attention. Patients who had developed PPS were more susceptible having undergone transfusion, and recurrence (38) is a common outcome. Although the research was not a direct comparison of closure versus non-closure, the clinical burden of PPS was presented, and the importance of preventive measures was emphasized, of which pericardial reconstruction is becoming more and more popular [11]. Equally, van Osch et al. have shown that PPS in 822 patients undergoing valve surgery was found in 14.5% and was linked with a tenfold increase in risk of reoperation due to tamponade at a one-year follow-up (20.9% vs. 2.5%) [5]. These results support the importance of surgery to lessen the effects of inflammatory sequelae, such as systematic pericardial reconstruction.

Recently, posterior pericardiotomy has received a newfound interest as an easy but effective supplement to the prevention of pericardial effusions and atrial fibrillation following cardiac surgery. Abdelaziz et al. used a meta-analysis of 25 randomized trials that involved 4467 patients, and they indicated that the use of posterior pericardiotomy reduced postoperative atrial fibrillation by nearly 50 percent (11.7% vs. 23.7%, OR 0.49, 95% CI 0.38–0.61). Moreover, odds ratios of 0.66, 0.32, 0.15, and 0.18 indicated that the procedure greatly reduced the occurrence of supraventricular tachycardia, early and late pericardial effusions, and tamponade. In addition to clinical efficacy, the posterior pericardiotomy had also been linked to reduced hospitalization, and hence it is cost-effective. Such results support the idea that pericardial surgery aimed at increasing the effectiveness of drainage can prevent the development of effusions as well as the inflammatory process that leads to PPS [20].

In a comprehensive systematic review, Yuan summarized the findings of 20 studies and used 661 patients who had an operation as a sample, revealing that PPS was identified in 18.3 ± 15.9 days after the procedure, most of which involved CABG and replacement of the mitral valve. In 12.6% of cases there was tamponade, and in 15% there was need of surgical drainage. The author highlighted that surgical trauma and cardiopulmonary bypass are strong stimulators of systemic inflammation and release of anti-heart autoantigens. Specifically, Yuan underscored that pericardial damage and non-closure increase the likelihood of recurring effusion and PPS, whereas surgical methods that prioritize controlled drainage and reconstruction should be prioritized [3].

### 3.2. Pericardial Substitutes: Patches, Biomaterials, and Meshes

Patches and biomaterials have been increasingly used when primary closure using autologous pericardium is not possible. Rego et al. highlighted that, in addition to offering a scaffold to regenerate site-specific tissues, extracellular matrix-based substances, including decellularized porcine small intestinal submucosa, decrease the inflammation and the formation of adhesion. Synthetic netting has been considered in randomized and observational studies, demonstrating reduced pericardial effusions and postoperative rates of atrial fibrillation as compared to no closure. Bioresorbable materials are also tested, with results showing reduced rates of pericardial effusions and postoperative atrial fibrillation [19].

Husain et al., among others, have noted that these substitutes have been able to safeguard grafts against sternal compression, as well as make re-entry easier during sternotomy. They have some major strengths, which are their biocompatibility and less inflammatory response. Additionally, Rego et al. showed the results of the RECON study that showed reduced complications with biologically active patches as compared to the pericardium left open. This was accompanied by the reduced incidence of pleuropericardial effusion, which is a significant risk factor of PPS [19,32].

Even clinical reviews, like that by Maranta et al., have highlighted the use of standardized methods of closure with modern substitutes as important. They mention that pericardial effusions are one of the most common postoperative complications, which commonly prelude PPS. It can be possible to break the cascade to PPS by decreasing the formation of effusion by sealing it with substitutes [1]. Thus, the use of pericardial substitutes is not only mechanical, but it also seems to have an effect on the inflammatory pathways.

### 3.3. Minimally Invasive or Modified Cardiothoracic Approaches (Table 3)

The autoimmune response of PPS is central to surgical trauma and cardiopulmonary bypass. To reduce this risk, minimally invasive and modified surgical procedures are thus being considered. Lehto and Kiviniemi determine that younger age, pleural incision, and valve or ascending aortic surgeries have a greater risk of PPS than isolated CABG. Less invasive procedures can help to minimize the immunogenic load by minimizing pericardial trauma and by limiting pleural openings [17].

Maranta et al. underscored the fact that PPS is often underestimated and is poorly described in clinical practice. They indicated that the incidence is highly diverse—including 9% and 21% in adults based on the criteria—and affected by the method of surgery. By minimizing pericardial manipulation, off-pump procedures and thoracoscopic procedures are hypothesized to reduce the risk of PPS, but comparative trials are not strong [1].

Mizuno et al. gave a warning on procedural alterations in relation to the epicardial device implantation. They reported a unique situation of localized constrictive pericarditis occurring 23 years following implantation of epicardial cardioverter-defibrillator patches that were densely fibrotic, thereby requiring resection of the fibrotic tissue to resume cardiac activity. Albeit uncommon, these long-term complications emphasize the possible negative sequelae of foreign material in the pericardium and the need to focus on the innovation of the procedure that reduces pericardial trauma [33].

New evidence indicates that risk stratification and patients’ selection are paramount. Yücel et al. proved that the presence of systemic inflammatory indicators, including the Systemic Inflammatory Response Index (SIRI) and Monocyte-to-Lymphocyte Ratio (MLR), can be used to identify patients at high risk of developing PPS to the severity that necessitates surgical drainage. They found that in their cohort of 150 patients, MLR values greater than 0.575 on postoperative day 7 were sensitive in 84%, and SIRI values greater than 3.34 were specific in 81%. The integration of these markers into the perioperative monitoring might enable customizability of the surgical plans, with minimally invasive plans given a higher priority in high-risk patients [34].

Native valve-sparing surgeries, although physiologically beneficial, seem to be associated with increased risk of PPS. Holst et al. analyzed the results of 91 patients who underwent native valve-sparing aortic valve surgery and discovered that 23% of patients developed PPS in the first postoperative period. Blood type O (OR 3.15, 95% CI 1.06–9.41), valve-sparing root replacement (OR 3.12, 95% CI 1.01–9.59), and peak postoperative C-reactive protein levels above 15 mg/dL (OR 4.27, 95% CI 1.05–17.29) were identified as independent risk factors by logistic regression. It is important to note that the overall combined effect of the variables raised the risk of PPS to up to 73%. These findings underscore the need to identify vulnerable subgroups in the modified surgical methods, specifically in younger patients who have technically challenging operations, and such data can support the use of more preventative measures in this group [35].

Pediatric patients can especially be susceptible to PPS. Heching et al. retrospectively studied 97 children, who had surgically closed secundum atrial septal defects and found a PPS occurrence of 28% during the first year after surgery. Echocardiogram on discharge showed a small pericardial effusion was a strong predictor of PPS, with a median onset of 8 days following surgery and 63 and 27 percent discharges with the syndrome and without the syndrome, respectively (*p* = 0.001). It indicates that even comparably minor congenital surgeries may lead to severe inflammation of the pericardium, and even the initial postoperative imaging data should be used to direct the surveillance and prevention [36].

Another high-risk group is the adults affected with congenital heart disease. Khor et al. compared 214 adult patients with congenital heart defects who were operated on and found that 22% of them developed postoperative pericarditis, the majority occurring after repair of atrial septal defects (82.7% of cases in the subgroup of shunt repair). The autologous pericardium was widely used in these patients, and logistic regression showed that younger age, male sex, and Asian race were the independent predictors. Even though the majority of cases were solved in 2–4 weeks, 8.5% returned. These results highlight the importance of surgical and preventive interventions specific to the population with congenital heart disease, in which the threat of inflammation continues throughout adult life [37].

**Table 3:** Type, technique, and extent of procedure in relation to postpericardiotomy syndrome occurrence. PPS: Postpericardiotomy Syndrome, CABG: Coronary Artery Bypass Grafting, AVR: Aortic Valve Replacement, MVR: Mitral Valve Replacement, ASD: Atrial Septal Defect.

Study (Year)	Type of Study	Aim of the Study	PPS Incidence by Procedure Type	Conclusions Regarding the Impact of Surgical Extent on the Incidence of PPS
Lehto et al. (2020) [17]	Review	Investigation of factors which are associated with PPS incidence, diagnosis, management, and prognosis	- Greater PPS incidence in extensive procedures - Valve surgery increases PPS risk compared to CABG (aortic > mitral) - AVR patients have higher PPS risk; MVR patients show lower or moderate risk. - Ascending aortic surgery shows the highest PPS incidence among adult procedures. - Urgent/emergency surgeries are associated with higher PPS occurrence	Εxtensive procedures with more myocardial damage are associated with increased PPS incidence
Maranta et al. (2022) [1]	Review	Presentation of an overview of PPS incidence, features, management, and knowledge gaps	- PPS occurs more frequently after aortic and aortic valve surgeries (26% vs. 7.9% after CABG and 8.3% after MVR). - Intraoperative pleural incision significantly increases PPS risk (HR 4.31; 95% CI 2.22–8.33). - More traumatic surgical procedures lead to a higher PPS incidence. - In pediatric patients, certain procedures, such as Fontan operation and ASD closure, are associated with higher PPS incidence	More traumatic surgical procedures which are associated with extensive pericardial manipulation lead to a stronger inflammatory response and higher PPS incidence
Holst et al. (2024) [35]	Retrospective single-center cohort study	Determination of the PPS incidence and identification of the perioperative predictors in patients undergoing native valve-sparing aortic valve surgery	- PPS developed in 21/91 patients (23%) after native valve-sparing aortic valve surgery. - 70/91 (77%) served as non-PPS controls. - Incidence was reported for the overall cohort and not stratified by individual valve-sparing surgical subtypes	More extensive valve-sparing procedures, particularly valve-sparing root replacement, were independently associated with an increased risk of PPS
Heching et al. (2015) [36]	Retrospective single-center observational cohort study	Determination of the PPS incidence after surgical closure of secundum ASDs in children and identification of the perioperative risk factors predictive of its development	- Among 97 pediatric patients, 27 (28%) developed PPS within the first postoperative year, while 70 (72%) did not develop PPS	- PPS was common after surgical ASD closure. - The study did not compare different cardiac surgery types but identified that the presence of a small pericardial effusion on discharge echocardiogram was associated with subsequent PPS
Khor et al. (2025) [37]	Retrospective observational cohort study	Evaluation of the incidence and perioperative predictors of postoperative pericarditis following cardiac surgery in adults with congenital heart disease	- Among 214, 47 (22.0%) developed postoperative pericarditis. - The majority (37/47, 78.7%) occurred within the first 7 postoperative days, and 4 (8.5%) experienced recurrent pericarditis. - Pericarditis was most frequently observed after shunt repair operations (27/47, 57.4%)—particularly ASD repair (24/29, 82.7%)—and also after AVR (10/29, 34.4%)	Postoperative pericarditis occurred most commonly in patients undergoing shunt repair, especially ASD repair with autologous pericardial patch, suggesting that procedures involving extensive pericardial manipulation/repair may be associated with a higher incidence of PPS

### 3.4. Pericardial Lavage and Local Drug Delivery Strategies

In addition to closure and substitutes, novel intraoperative techniques, including pericardial lavage and local drug delivery, have also been suggested to minimize postoperative inflammation. Although no large-scale randomized data exist, there are initial reports of benefit in early experimental and small clinical trials. As Maranta et al. found, effusions caused by inflammatory events are antecedents to PPS; it was conceivable that pericardial space clearance of inflammatory mediators might prevent the onset of the syndrome [1].

Another idea promoted by Lehto and Kiviniemi is that in high-risk populations, especially during valve or aortic surgery, PPS may occur frequently (as high as 20 percent), and aggressive prevention is recommended. There has been a pilot investigation of local delivery of anti-inflammatory therapies like corticosteroids or biologic agents into the pericardial space, but there are concerns about the risk of infection and local toxicity [17].

Rapid pericardial fluid identification and drainage strategies are clearly defined in the literature on trauma surgery and provide information that is applicable in elective cardiac surgery. Ingraham and Sperry provided the evidence of subxiphoid pericardial window and lavage as per penetrating cardiac injury. However, these methods emphasize the usefulness of direct pericardial drainage in the reduction in tamponade as well as in the prevention of inflammation, although it is intended to be used in cases of emergent diagnosis and decompression. Applied to the cardiac surgery context, these strategies indicate that proactive lavage and de-compressions would theoretically decrease the occurrence of the PPS due to the prevention of the continued occurrence of the inflammatory effusions [38].

New approaches can combine mechanical and pharmacological protection by incorporating biomaterials with drug-eluting characteristics. As an illustration, bioresorbable nets that are soaked in anti-inflammatory substances would theoretically block the collection of effusion as well as the autoimmune cascade. As an experimental approach, these approaches nonetheless become a promising future of PPS prevention.

## 4. Biomarkers and Risk Stratification

### 4.1. Predictive Biomarkers

Studies on PPS have also been growing on examining laboratory and imaging biomarkers that can predict the most likely patients to develop clinically meaningful disease (Table 4).

NLR has become a possible indicator of systemic inflammation following cardiac surgery. Sevuk et al. presented results of a study of 172 patients undergoing an elective coronary artery bypass grafting and revealed that postoperative, but not preoperative, NLR was significantly correlated with PPS. Patients with PPS had elevated postoperative white blood cell counts and NLR values with a cutoff of 8.34 favoring the occurrence of PPS with a 60 to 59 sensitivity/specificity, respectively. In addition, postoperative NLR was independently related to PPS (OR 3.3, 95% CI 1.56–7.01), indicating that this convenient biomarker may be used to identify the risk of an early eventuality [21].

Another similar research connects inflammatory biomarkers to the overlap between PPS and postoperative atrial fibrillation (POAF). Sevuk et al. found that patients with POAF were almost twice as likely to develop PPS than patients without arrhythmia (61.7% vs. 45.8%, *p* = 0.04). These results validate the hypothesis that the two syndromes share systemic and pericardial inflammation as a common denominator and that inflammatory biomarkers can be used to assist in the surveillance of joint complications [39].

Despite advances in novel biomarkers, classical inflammatory markers remain clinically relevant. CRP is commonly elevated in patients with PPS and has consequently been integrated into diagnostic criteria. Tamarappoo and Klein noted that the European Society of Cardiology guidelines define PPS as the presence of at least two of five diagnostic criteria, one of which is pleural effusion accompanied by elevated CRP levels. They further emphasized the role of echocardiography and cardiac magnetic resonance imaging in detecting pericardial thickening, inflammation, and effusion, which can serve as imaging adjuncts to biomarker-guided risk assessment. Consequently, the combination of CRP with imaging criteria can strengthen both the diagnosis of PPS and the prediction of associated risks [40].

In addition to circulating blood markers, pericardial fluid analysis has been investigated to predict. Mitu et al. analyzed 10 studies, the results of which indicated that the amount of pericardial fluid biomarkers, including IL-6, mitochondrial DNA, myeloperoxidase, and natriuretic peptides, was associated with POAF, a complication that is often related to PPS. These molecules are indicative of elevated levels of inflammatory and oxidative stress, atrial remodeling, and myocardial strain. As an example, high levels of pericardial brain natriuretic peptide (BNP) and atrial natriuretic peptide (ANP) were linked to the presence of impaired left ventricular functioning, and IL-6 was also consistently high in the patients with postoperative arrhythmias. Despite the small population sizes of the majority of the studies, these results indicate that personalized risk models can be improved by sampling pericardial fluid perioperatively [22].

The role of immune dysregulation is also further emphasized by systematic reviews of determinants of PPS. Low preoperative IL-8 and high postoperative complement activation products were linked to the risk of PPS, according to Van Osch et al., suggesting that both excessive postoperative inflammation and impaired immune priming at baseline are risk factors. Besides this, a decrease in preoperative platelet and hemoglobin levels and perioperative transfusion were observed to have a higher risk of PPS, indicating that both immunologic and hematologic biomarkers could be used in predictive modeling [5].

These biomarker findings are supported by epidemiologic data, which puts clinical risk into perspective. In their prospective study, Lehto et al. found that PPS necessitating medical care was found to be 8.9% among 688 patients undergoing isolated CABG, where transfusion of red blood cell units and renal insufficiency functioned as independent predictors. Notably, PPS reoccurred in 38% of instances, and greater body mass index forecasted relapse. Such clinical indicators in conjunction with lab markers like NLR and CRP may be the basis of composite risk scales [11].

Lastly, population modifiers of PPS incidence have been determined by use of registry-based studies. According to Lehto et al., PPS was more prevalent after aortic valve replacement, mitral valve replacement, and ascending aorta surgeries compared to CABG and was linked with a higher mortality of 1.78 times at one year. These risks related to procedures underscore the value of integrating both the variables related to surgery with biomarker data to achieve a holistic risk stratification [9].

### 4.2. Personalized Prevention Approaches

The discovery of predictive biomarkers has already opened avenues toward individualized preventive strategies. Patients with elevated postoperative NLR or high CRP levels could undergo closer echocardiographic surveillance and be initiated earlier on anti-inflammatory prophylaxis, such as colchicine or NSAIDs. Conversely, patients with low inflammatory biomarker profiles may be spared unnecessary pharmacological interventions [4,15].

As shown by Lehto et al., severe PPS requiring invasive intervention after surgical aortic valve replacement was associated with a twofold increase in mortality (HR 2.01, 95% CI 1.03–3.91). Given that extreme forms of PPS are linked to excess mortality, the identification of predictive biomarkers could facilitate early recognition of patients at risk for aggressive disease, enabling individualized follow-up and more intensive preventive treatment strategies [10].

Novel imaging modalities can also contribute to personalized care. Cardiac magnetic resonance imaging and echocardiography aid in diagnosis and allow quantification of pericardial effusion burden, pericardial thickening, and inflammatory activity. Tamarappoo and Klein proposed that imaging-based risk scores, combined with inflammatory biomarkers such as CRP or NLR, could be used to stratify patients with PPS into low-, intermediate-, and high-risk categories [40].

Specific prevention methods, including sex- and age-specific methods, might be justified. Lehto et al. discovered that the incidence of PPS among women was higher compared to the number of cardiac surgeries, whereas younger patients had the highest absolute rates of hospitalization because of PPS. In this way, it can be assumed that demographic variables can be combined with biomarkers to develop personalized prevention initiatives [41].

There is also a growing shift toward integrating pericardial fluid biomarkers with systemic measurements. As suggested by Mitu et al., levels of IL-6 and natriuretic peptides in pericardial fluid correlate with their corresponding blood concentrations, representing a feasible approach for perioperative risk prediction. If confirmed in larger cohorts, such biomarkers could guide risk-adjusted use of anti-inflammatory agents or targeted biologic therapies [22].

Finally, Wamboldt et al. emphasized that conventional pharmacological prevention interventions, like corticosteroid usage, have been found to be varied in their effectiveness and still have risks. Thus, personalization based on biomarkers would potentially aid in identifying subgroups that would prefer the use of corticosteroid or biologic treatment but without exposing the low-risk patient to needless exposure [42].

## 5. Conclusions

PPS is a common and clinically significant complication after cardiac surgery, with a substantial impact on patient outcomes, readmission rates, and long-term prognosis. Accumulating evidence indicates that prevention is most effective when a multimodal strategy is applied, integrating pharmacological prophylaxis, surgical innovation, and structured risk stratification rather than relying on a single intervention.

Colchicine continues to be the main preventive pharmacological agent, as evidenced by reliable randomized trials [12,13,14,17,24,25,26,27,28]; however, its gastrointestinal intolerance significantly restricts its widespread application. Despite robust randomized evidence supporting colchicine for PPS prevention, important sources of heterogeneity and bias should be considered when interpreting pharmacologic data. Variability in colchicine dosing, timing of initiation (pre- vs. post-operatively), PPS definitions, and follow-up duration contributes to between-study heterogeneity, while gastrointestinal intolerance and treatment discontinuation may introduce attrition bias.

Evidence supporting NSAIDs for PPS prevention is limited [15,16]. Therefore, no definitive conclusions can be drawn regarding their prophylactic efficacy, but only with respect to the treatment of PPS, for which the supporting evidence is historically well established.

Emerging targeted therapies, particularly IL-1 inhibitors, show promise for patients at high risk or with refractory disease, although further evidence is required before their routine implementation can be recommended.

From a surgical perspective, techniques such as posterior pericardiotomy and pericardial closure using biomaterials constitute evidence-based approaches that significantly reduce pericardial effusion, atrial fibrillation, and cardiac tamponade. Given that pericardial trauma is thought to trigger the autoimmune mechanisms underlying PPS, minimally invasive surgical strategies may further mitigate disease development. These approaches appear particularly relevant in high-risk populations, including pediatric patients, individuals undergoing valve-sparing procedures, and those with congenital heart disease.

The interpretation of surgical strategies for the prevention of PPS is constrained by substantial heterogeneity and methodological limitations. Evidence regarding pericardial reconstruction and closure is derived predominantly from observational studies and expert consensus [19,20,32], rendering the reported associations susceptible to confounding by indication, surgeon preference, and center-specific practice patterns. Variability in surgical techniques, the materials used, and outcome definitions further contributes to interstudy heterogeneity. While posterior pericardiotomy is supported by randomized clinical studies demonstrating consistent reductions in pericardial effusions and atrial tachyarrhythmias, extrapolation to PPS prevention should be undertaken with caution, as many trials were not designed with PPS as a primary endpoint. Overall, surgical interventions appear to mitigate inflammatory sequelae through improved drainage and reduced pericardial injury; however, causal inference remains limited outside randomized settings.

Perhaps the most promising advance lies in biomarker-based risk prediction. Readily available parameters such as NLR and CRP, when combined with imaging and demographic variables, may help identify patients at higher risk for PPS and support more individualized perioperative management. This approach could be further refined through the incorporation of biomarkers, including IL-6 and natriuretic peptides, which provide mechanistic insights into local inflammatory and myocardial stress responses. However, interpretation of biomarker data requires caution, as most supporting evidence derives from observational cohorts characterized by heterogeneous surgical populations, variable timing of sampling, and inconsistent PPS definitions [5,9,11,21,38]. Biomarkers such as postoperative NLR or CRP may reflect the magnitude of surgical trauma, systemic inflammation, or perioperative complications rather than PPS-specific pathophysiology, raising concerns regarding reverse causality. Moreover, many associations are closely collinear with illness severity, transfusion burden, renal dysfunction, or postoperative atrial fibrillation, thereby limiting independent predictive value. Pericardial fluid biomarkers, while biologically appealing, are supported mainly by small, single-center studies and remain vulnerable to selection bias and confounding by indication [22]. Collectively, these considerations suggest that biomarkers should be integrated as components of composite clinical risk models rather than used as standalone tools, pending prospective validation and standardized biomarker-driven risk stratification strategies.

Therefore, future research should focus on validating biomarker-driven risk models in large, prospective cohorts and assessing their effectiveness in guiding preventive interventions. Multidisciplinary collaboration among cardiologists, cardiac surgeons, and immunologists will be essential to advance this field.

Ultimately, the most effective strategy to reduce the burden of PPS and improve outcomes after cardiac surgery is a shift toward individualized, evidence-based prevention that integrates pharmacological, surgical, and biomarker-guided interventions.

## Figures and Tables

**Table 1:** Clinical studies assessing the efficacy and effect estimates of colchicine in the prevention of postpericardiotomy syndrome. PPS: Postpericardiotomy Syndrome, RR: Relative Risk, POAF: Postoperative Atrial Fibrillation, NR: Not Reported, AF: Atrial Fibrillation, GI: Gastrointestinal.

Study, Year	Study Design	Study Objective	Key Findings	Effect Size
Imazio et al., 2010 (COPPS trial) [12]	Multicenter, randomized, double-blind, placebo-controlled trial	To evaluate whether colchicine administration after cardiac surgery reduces the incidence of PPS	Colchicine significantly reduced the incidence of PPS compared with placebo. PPS occurred in 8.9% of patients receiving colchicine versus 21.1% in the placebo group	RR reduction 57% (colchicine vs. placebo)
Imazio et al., 2014 (COPPS-2 trial) [17]	Randomized, double-blind, placebo-controlled clinical trial	To assess whether preoperative initiation of colchicine reduces the incidence of PPS and POAF	Preoperative colchicine significantly reduced the incidence of PPS and POAF compared with placebo. However, colchicine was associated with a higher rate of gastrointestinal intolerance, leading to drug discontinuation in 21% of patients versus 5% in the placebo group	RR reduction for PPS ~50% (colchicine vs. placebo)
Agarwal et al., 2015 [13]	Meta-analysis of randomized clinical data	To evaluate colchicine effectiveness for prevention of recurrent pericarditis and PPS	Reduced PPS incidence at 1 year: 13.2% vs. 25.8% (colchicine vs. placebo) with increased adverse events (e.g., diarrhea: 12.5% vs. 8.5%, respectively)	RR 0.56 (95% CI 0.42–0.76), colchicine vs. placebo
Verma et al., 2015 [14]	Systematic review and meta-analysis of randomized controlled trials	To evaluate colchicine effectiveness across cardiac disease	Relative decrease in PPS incidence by ~50% and reduction in peri-procedural AF; discontinuation due to GI intolerance ~10%	NR
Mashayekhi et al., 2020 [24]	Double-blind randomized placebo-controlled clinical trial	To evaluate colchicine for prevention of PPS	PPS reduced by about half: 12.1% vs. 21.6% (colchicine vs. placebo), without serious complications	NR
Amoli et al., 2015 [25]	Randomized placebo-controlled trial	Colchicine vs. placebo for treatment of pericardial effusion after open-heart surgery	No significant reduction in pericardial effusion volume or PPS-related outcomes.	NR
Meurin et al., 2015 [26]	Multicenter, double-blind randomized controlled trial	To assess colchicine for prevention/treatment of postoperative pericardial effusion	Colchicine did not significantly reduce postoperative pericardial effusion or PPS-related endpoints	NR
Lutschinger et al., 2019 [27]	Meta-analysis	To assess colchicine efficacy in pericarditis and PPS	Colchicine significantly reduced PPS incidence; benefit dependent on early initiation	PPS: Pooled RR 0.57
Pan et al., 2023 [28]	Randomized controlled trial	To evaluate low-dose colchicine for PPS prevention and myocardial protection	Significant reduction in PPS incidence and inflammatory/myocardial injury markers	PPS: RR 0.18

**Table 2:** Surgical interventions and outcomes in postpericardiotomy syndrome prevention. AF: Atrial Fibrillation, LoHS: Length of Hospital Stay, NR: Not Reported, POAF: Postoperative Atrial Fibrillation, SVT: Supraventricular Tachycardia.

Study, Year	Study Design/Population	Surgical Intervention & Comparator	Key Outcomes	Effect Size
Rego et al., 2022 [19]	Expert consensus	Pericardial reconstruction/closure vs. non-closure after cardiac surgery	Reported associations with reduced pericardial adhesions, postoperative effusions, atrial fibrillation, bleeding complications, length of hospital stay, and readmissions; no hemodynamic compromise reported with patch-based closure techniques	NR
Husain et al., 2016 [32]	Technical report	Anatomical approximation of the upper pericardium using hemostatic clips vs. conventional non-closure	The technique required minimal additional operative time, allowed protection of bypass grafts and great vessels, facilitated safe re-entry during reoperation, and was not associated with early postoperative complications such as tamponade; reduced pericardial adhesions were qualitatively reported	NR
Abdelaziz, 2023 [20]	Systematic review and meta-analysis of 25 randomized controlled trials	Posterior pericardiotomy performed at the time of cardiac surgery vs. standard surgical approach without pericardiotomy	Significant reduction in POAF and SVT, accompanied by a marked decrease in pericardial effusions and cardiac tamponade. Posterior pericardiotomy was also associated with improved postoperative drainage, shorter LoHS, and reduced need for reintervention	- POAF: OR 0.49 (95% CI 0.38–0.61) - SVT: OR 0.66 - Early pericardial effusion: OR 0.32 - Late pericardial effusion: OR 0.15 - Cardiac tamponade: OR 0.18
Yuan, 2020 [3]	Review	Surgical approaches with pericardial closure or controlled drainage vs. non-closure (descriptive, no formal comparator)	Timing of PPS onset after surgery; incidence of pericardial effusion and tamponade; need for medical or surgical drainage; association of pericardial injury and non-closure with recurrent effusions and PPS; emphasis on surgical trauma and cardiopulmonary bypass as triggers of systemic and local inflammation	NR

**Table 4:** Laboratory, Imaging, and Clinical Biomarkers Associated with Postpericardiotomy Syndrome (PPS). NLR: Neutrophil-to-Lymphocyte Ratio, CABG: Coronary Artery Bypass Graft, POAF: Postoperative Atrial Fibrillation, CRP: C-Reactive Protein, MRI: Magnetic Resonance Imaging, NSAIDs: Nonsteroidal Anti-Inflammatory Drugs, IL-6: Interleukin-6, BNP: B-type Natriuretic Peptide, NT-proBNP: N-Terminal pro-B-type Natriuretic Peptide, BMI: Body Mass Index, AVR: Aortic Valve Replacement, MVR: Mitral Valve Replacement.

Biomarker/Factor Type	Study, Year	Population/Design	Main Findings Related to PPS	Clinical Implication
Inflammatory biomarker—NLR	Sevuk et al., 2016 [21]	Retrospective cohort of elective on-pump CABG patients	Elevated postoperative NLR independently predicted PPS (cut-off 8.34; OR 3.3), whereas preoperative NLR was not predictive	Postoperative NLR may assist in early identification of patients at increased risk for PPS after CABG
Clinical factor—new-onset POAF	Sevuk et al., 2016 [38]	Retrospective cohort of isolated on-pump CABG patients with and without POAF	Early PPS occurred significantly more often in patients with POAF than without (61.7% vs. 45.8%, *p* = 0.04), and POAF was independently associated with increased early PPS risk (OR 1.9; 95% CI 1.03–3.5; *p* = 0.04)	Patients developing POAF after CABG should be closely monitored for early PPS due to elevated risk
Clinical and imaging markers summarizing PPS features	Tamarappoo & Klein, 2016 [39]	Literature review of PPS across cardiothoracic surgery cohorts	PPS occurs after cardiothoracic surgery and is characterized by fever, pleuritic pain, pericardial/pleural effusions, and elevated CRP; echocardiography and cardiac MRI aid diagnosis and monitoring.	Recognition of typical clinical features and use of imaging/CRP can improve early PPS diagnosis and guide anti-inflammatory treatment with NSAIDs/colchicine
Pericardial fluid biomarkers (IL-6, mitochondrial DNA, myeloperoxidase, BNP, NT-proBNP)	Mitu et al., 2025 [22]	Systematic review of studies evaluating pericardial fluid biomarkers and their association with POAF after cardiac surgery	Elevated pericardial fluid IL-6, mitochondrial DNA, and myeloperoxidase were associated with increased risk of POAF, suggesting an inflammatory pericardial milieu that may also relate to PPS outside arrhythmia context	Pericardial fluid inflammatory biomarkers may help identify patients at higher risk for inflammatory postoperative complications, potentially including PPS, prompting closer monitoring or early anti-inflammatory strategies
Clinical risk factors (BMI, pulmonary disease treatment)	Van Osch et al., 2017 [5]	Single-center retrospective cohort of nonemergent valve surgery patients	PPS occurred in 119/822 (14.5%); higher BMI was associated with lower PPS risk (OR 0.94 per point), and preoperative treatment for pulmonary disease without corticosteroids was linked to increased PPS risk (OR 2.55); PPS patients had more reoperations for tamponade (20.9% vs. 2.5%)	Recognition of specific preoperative risk factors (pulmonary disease without steroids, lower BMI) may help identify valve surgery patients at increased risk for PPS and tamponade, informing monitoring and management strategies
Clinical and procedural risk factors (red blood cell transfusion, renal insufficiency, diabetes)	Lehto et al., 2015 [11]	Retrospective cohort of adults undergoing isolated CABG surgery with long-term follow-up	PPS occurred in 61/688 (8.9%) patients; independent predictors of PPS were renal insufficiency and ≥1 unit of red blood cell transfusion, whereas diabetes was associated with lower PPS risk; median time to diagnosis was 21 days and recurrence occurred in 38%	Recognition of perioperative bleeding requiring transfusion and renal dysfunction as PPS risk factors may help tailor monitoring and preventive strategies after CABG
Procedural risk factors (type of cardiac surgery: AVR, MVR, aortic surgery vs. CABG) and age	Lehto et al., 2018 [9]	Nationwide retrospective registry cohort of consecutive adult cardiac surgery patients including CABG, AVR, MVR, and ascending aortic surgery. Only PPS episodes leading to hospital admission or contributing to death were included	PPS occurred in 493/28,761 patients; incidence was significantly higher after AVR (HR 1.97), MVR (HR 1.62), and aortic surgery (HR 3.06) compared with CABG; increasing age was associated with lower PPS risk; PPS was linked with higher 1-year postoperative mortality (adjusted HR 1.78)	Recognition that PPS incidence varies by procedure type and is associated with increased mortality highlights the importance of targeted surveillance and early management, especially after valvular and aortic operations

## Data Availability

Data supporting these findings are available within the article.

## Abbreviations

The following abbreviations are used in this manuscript:

PPS	Postpericardiotomy syndrome
NSAIDs	Nonsteroidal Anti-inflammatory Drugs
NLR	Neutrophil-to-Lymphocyte Ratio
CRP	C-Reactive Protein
IL	Interleukin
NLRP3	NLR family pyrin domain containing 3
COPPS	Colchicine for the Prevention of the Postpericardiotomy Syndrome
OR	Odds Ratio
CI	Confidence Interval
CABG	Coronary Artery Bypass Grafting
SIRI	Systemic Inflammatory Response Index
MLR	Monocyte-to-Lymphocyte Ratio
POAF	Postoperative Atrial Fibrillation
BNP	Brain Natriuretic Peptide
ANP	Atrial Natriuretic Peptide
